# A Novel Coronavirus Outbreak from Wuhan City in China, Rapid Need for Emergency Departments Preparedness and Response; a Letter to Editor 

**Published:** 2020-02-02

**Authors:** Mostafa Alavi-Moghaddam

**Affiliations:** 1 Emergency Medicine Department, Imam Hossein Hospital, Shahid Beheshti University of Medical Sciences, Tehran, Iran

Dear Editor 

On 31 December 2019, Chinese authorities reported the increase in incidence of severe pneumonia in Wuhan city, Hubei province of China. One week later, on January 7^th^, they confirmed that they had identified a new coronavirus, which is a family of microRNA respiratory viruses including the common cold, and viruses such as Severe Acute Respiratory Syndrome (SARS) and Middle East Respiratory Syndrome (MERS). This new virus was temporarily named “2019-nCoV”. Wuhan city is a major international transport hub. This report to World Health Organization (WHO), raised global public health concern because this is the third coronavirus –associated acute respiratory illness outbreak. 

Currently, up to the date of submitting this letter, 4593 cases of 2019-nCoV infections have been confirmed globally, both in China (4537 have been confirmed, 976 of them presented with severe disease and 106 died) and outside of China (56 confirmed in 14 countries.). WHO risk assessment of 2019-nCoV infection is Very High in China and High in other countries (1).

Although 2019-nCoV has not been included in the WHO blueprint list of priority diseases yet, MERS CoV, and SARS –CoV, which are already included in this list, are both coronaviruses that have led to global outbreaks in 2003 and 2012, respectively. 

The specific source and the exact primary mode of transmission of 2019-nCoV to humans remain unknown. The clinical features and laboratory and radiological abnormalities with 2019-nCoV infections are not specific and are similar to other respiratory tract infections.

Adults and pediatrics who acquire a 2019-nCoV infection can show a spectrum of respiratory illness severity, from asymptomatic to mild, moderate or severe disease. The severe disease manifests as severe acute respiratory infection (SARI) or severe pneumonia, Acute Respiratory Distress Syndrome (ARDS), sepsis and septic shock. Patients with pre-existing medical comorbidities develop a more severe disease and have higher mortality rates compared to patients who do not have any comorbidity.

Clinical care of patients with suspected 2019-nCoV should focus on early recognition, immediate isolation (separation), implementation of appropriate infection prevention and control (IPC) measures and provision optimized supportive care. 

At the triage of an Emergency room, early recognition of suspected patients allows for timely initiation of IPC. 2019-nCoV should be considered as a possible etiology of influenza like illness (ILI) under certain situations according to case definitions of WHO (2). Both the health care worker (HCW) and the suspected case of acute respiratory illness (ALI) should wear a medical mask and the patient should better be directed to a separate area, an isolation room if available. Otherwise, keep a distance of at least one meter between suspected patients and other patients. Instruct all suspected patients to cover their nose and mouth during coughing or sneezing with tissue or flexed elbows for protecting others. Those with mild or moderate clinical presentations of the 2019-nCoV infection may not require hospitalization, unless there is concern of rapid deterioration.

 All patients discharged to go home directly from fast track in emergency room should be instructed to consider IPC measures and to return hospital if their symptoms worsen (3). Patients with severe illness, who are admitted to the emergency ward, should be transferred to the floor and if available to the ICU ward as soon as possible. As long as they stay in emergency ward, they should be placed in single rooms or grouped together with those who have the same etiological or clinical diagnosis. Limit patient movement within the center and ensure that patients wear medical masks when outside their rooms. HCW should perform hand hygiene after contact with respiratory secretions. Droplet and contact precautions prevent direct or indirect transmission of the disease from contact with contaminated surfaces or equipment. HCW should use personal protective equipment (PPE) including medical mask, eye protection, gloves and gown, when entering the room and remove PPE when leaving. If equipment needs to be shared among patients, they should be cleaned and disinfected after each patient’s use. HCW should apply airborne precautions when performing an aerosol generating procedure (i.e. open suctioning of respiratory tract, intubation, bronchoscopy, cardiopulmonary resuscitation) (4).

HCW should immediately provide supplemental oxygen therapy for patients with SARI and respiratory distress, hypoxemia or shock. Oxygen therapy flow rate should be aimed at Spo2>=90%, Spo2>=92-95% and Spo2>=94%, in non-pregnant, pregnant and children, respectively. HCW should recognize severe hypoxemic respiratory failure when a patient with respiratory distress is failing standard oxygen therapy. High-flow nasal oxygen (HFNO) or non-invasive ventilation (NIV) should be used in selected patients with hypoxemic respiratory failure. Hypoxemic respiratory failure due to ARDS among these patients commonly results from intrapulmonary ventilation-perfusion mismatch or shunt and usually requires mechanical ventilation. Thus, rapid sequence intubation should be performed using airborne precautions. Implementation of mechanical ventilation using lower tidal volumes (4-8 ml/kg predicted body weight) and higher positive end-expiratory pressure (PEEP) is suggested. Patients with SARI should be treated cautiously with intravenous fluids when there is no evidence of shock, because aggressive fluid resuscitation may worsen oxygenation. For resuscitation of septic shock in adults, at least 30 ml/kg of isotonic crystalloid should be infused in the first 3 hours of shock identification and in children rapid bolus of 20 ml/kg as loading dose and up to 40-60 ml/kg of isotonic crystalloid infusion in the first hour of shock identification is needed. Vasopressor should be administered when shock persists during or after fluid resuscitation. If signs of poor perfusion persist despite reaching mean arterial pressure (MAP) target (i.e. >65 mmHg) with fluids and vasopressor, consider administering an inotrope such as dobutamine. Empiric antimicrobials should be initiated within one hour of identification of sepsis to treat all likely pathogens causing SARI. Empiric antibiotic treatment should be based on the clinical diagnosis of severe pneumonia or sepsis, local epidemiology and susceptibility data as well as treatment guidelines. If influenza is also a concern and there is a local circulation of influenza virus, a neuraminidase inhibitor should be adjoined to empiric therapy. Empiric antibiotic therapy should be de-escalated on the basis of microbiology results and clinical judgment. Systemic corticosteroids should not be routinely adding to therapy unless indicated for another reason. Collection of clinical specimens for laboratory diagnosis is suggested in early outbreak period and after that it is only advised for investigational purposes. If laboratory diagnosis is considered, serology is recommended only when RT-PCR is not available. Otherwise, HCW should collect specimens from both the upper respiratory tract and lower respiratory tract for testing 2019-nCoV via RT-PCR (5).

At the time being, emergency preparedness and response for providing appropriate care to the patients suspected to coronavirus-associated acute respiratory illness (above-mentioned plans) should be developed and implemented in the emergency departments, as the frontline of treating human infections of 2019-nCov in the hospitals.
